# From Learning to Practice: Support Workers' Experiences With the TRAIN‐ID Training Programme to Strengthen Health Support for People With Intellectual Disabilities

**DOI:** 10.1111/jar.70326

**Published:** 2026-10-20

**Authors:** Kim Nijhof, Fleur H. Boot, Kirsten E. Bevelander, Elske van Oostaijen, Jenneken Naaldenberg, Geraline L. Leusink

**Affiliations:** ^1^ Department of Primary and Community Care Radboud University Medical Center Nijmegen the Netherlands; ^2^ Academic Collaborative Intellectual Disability and Health ‐ Sterker op Eigen Benen (SOEB) Nijmegen the Netherlands; ^3^ HAN University of Applied Sciences, STERKplaats Nijmegen the Netherlands; ^4^ The Dutch National Professional Association for Support Workers (BPSW) Utrecht the Netherlands

**Keywords:** health support, intellectual disabilities, Kirkpatrick framework, support workers, training evaluation

## Abstract

**Background:**

Support workers play a key role in everyday health support for people with intellectual disabilities. This study aimed to explore how training (the TRAIN‐ID programme) can strengthen support workers' competencies to meet the health needs of people with intellectual disabilities.

**Method:**

This qualitative study explored 26 support workers' experiences with participating in the training programme through written reflections, focus groups immediately post‐training and interviews three months later. Data were analysed using template analysis informed by the Kirkpatrick framework.

**Results:**

Positive experiences with the programme related to observing, discussing and following up health problems in practice. Furthermore, analysis indicates concrete pathways linking learning to intentions for behavioural change and perceived impact.

**Conclusions:**

Training programmes that recognise support workers' roles in health support strengthen support workers' ability to identify and respond to health concerns, thereby helping to address educational gaps and improving everyday health support for people with intellectual disabilities.

## Highlights

- We developed a training programme called TRAIN‐ID. In the TRAIN‐ID training, support workers learn to better support the health of people with intellectual disabilities.
- Support workers said the TRAIN‐ID training was useful in their daily work. They learned more about health and how they can help people with intellectual disabilities stay healthy.
- After the training, support workers noticed changes in people's health more often. They also talked more about health with people with intellectual disabilities and with healthcare professionals.
- Support workers could not always use what they had learned. Being very busy and difficulties working together sometimes made this hard. When studying training programmes, researchers should therefore also look at what happens in the workplace.

## 1 Introduction

People with intellectual disabilities experience poorer health outcomes, including higher rates of chronic conditions and premature mortality, compared with the general population (McMahon and Hatton 2021; Baksh et al. 2023; Heutmekers et al. 2026; Rydzewska et al. 2025). Their healthcare needs are often complex, as communication difficulties and limited health literacy can hinder the recognition, interpretation and articulation of health problems (Mastebroek et al. 2016; Evenhuis et al. 2000). In the Netherlands, care is delivered across a range of settings, from independent living with occasional support to residential facilities providing 24‐h care (Breuer et al. 2024; Schalock et al. 2021; Wullink et al. 2007). Within these care contexts, support workers provide daily assistance and are therefore crucial in addressing the health needs of people with intellectual disabilities (Nijhof et al. 2024; Kroezen et al. 2018).

Support workers' initial education, which focuses on social care, daily living support and participation, often prepares them insufficiently for health‐related responsibilities. Supporting clients with their health requires specific competencies, such as timely identification of health concerns and addressing them appropriately, which can help prevent misinterpretation of discomfort and delayed diagnoses (Ervin et al. 2014; McKenzie 2013; Salvador‐Carulla et al. 2015; Northway et al. 2017). To meet the complex health needs of people with intellectual disabilities, additional training is necessary to equip support workers with the required knowledge and skills (O'Leary et al. 2018; Emerson et al. 2016; Barratt et al. 2023; McShea et al. 2016).

Current training programmes for support workers focus primarily on specific topics and competencies like healthy living, communication with healthcare professionals and healthy ageing (Northway et al. 2017; Overwijk et al. 2022; Vlot‐van Anrooij et al. 2020; Ziviani et al. 2004; García et al. 2020; Acton et al. 2023). These programmes provide an important foundation for health‐related support. However, because support workers are central in the continuous, day‐to‐day health support for people with intellectual disabilities, there is a need to further strengthen how health support is integrated into support workers' everyday practice. This includes promoting healthy living, recognising early signs of health problems and initiating appropriate follow‐up actions (Nijhof et al. 2024; Windley and Chapman 2010).

To ensure that training enhances support workers' role in health support effectively, it is important to assess how it translates into practice. Training is widely assumed to be beneficial, but evidence on its impact on support workers' behaviour and on care outcomes for people with intellectual disabilities remains limited (Rose and Gallivan 2019). When support workers see the impact of their health‐supporting behaviour on clients' well‐being, this can reinforce their motivation and support the integration of health support practices in daily care (Hatton et al. 1999). Evaluation frameworks must be used to move from describing solely what is taught in training programmes towards understanding whether training is effective in practice. Originally developed to evaluate workplace training in industry, the Kirkpatrick framework has since been applied in research on health professions education, including the evaluation of staff training in disability services (Allen et al. 2022; Smidt et al. 2009). The framework offers a structured approach to assessing training effectiveness across four levels: reaction (participants' satisfaction), learning (changes in knowledge, skills, awareness or confidence), behaviour (changes in professionals' behaviour) and results (impact on organisational or care outcomes) (Kirkpatrick and Kirpatrick 2011). This framework is particularly useful for evaluating training in complex care contexts because it explicitly addresses the transfer of learning into professional behaviour and its perceived impact on practice.

This study aims to contribute to a deeper understanding of how training can strengthen support workers' competencies to meet the health needs of people with intellectual disabilities in everyday practice by addressing the following research question: *What are support workers' experiences with a new training programme on health support for people with intellectual disabilities and how do they perceive its impact on providing health support, as evaluated through the Kirkpatrick framework?*

## 2 Methods

### 2.1 Research Design

Guided by qualitative approaches to evaluate training (Patton 2014), we adopted a qualitative research design to assess support workers' experiences with TRAIN‐ID, a three‐day training programme about health support for people with intellectual disabilities. Data were collected through written feedback during the training, focus group discussions (FGD) immediately post‐training and interviews three months post‐training. The Ethical Review Board of Radboud University Medical Center assessed this study (Registration number 2024‐17091) and waived the requirement for a full review according to the Dutch Medical Research with Human Subjects Law (*Wet Medisch‐wetenschappelijk Onderzoek met mensen* (WMO)). We followed the ethical principles of the Declaration of Helsinki and General Data Protection Regulations.

### 2.2 Training Programme

The training programme, developed prior to this study and based on findings from previous scientific research (Nijhof et al. 2024), was pilot‐tested in this study with two participant groups. An intellectual disability physician and lecturer in intellectual disability medicine (FB) delivered the training. Two experts‐by‐experience, both with mild intellectual disability, served as co‐trainers on the second day, providing participants the opportunity to practice skills related to motivational interviewing. The training format emphasised active learning methods, including interactive exercises, case discussions and exchange of experiences among participants. This approach was chosen to align with the practice‐oriented nature of support workers' role and to connect learning directly to everyday care situations. Table 1 provides an overview of the training programme's focus and learning objectives.

**TABLE 1:** Focus and learning objectives per training day of the TRAIN‐ID training programme about health support for people with intellectual disabilities.

Day	Training focus	Learning objectives
1	Understanding and observing health of individuals with intellectual disabilities	The participant: knows the essential health characteristics of people with intellectual disabilities. understands the importance of getting to know the client to promote that client's health. knows how to observe and identify health problems. can assess the quality of their observations. can observe their client's behaviour and health situation and identify changes. can tailor the care plan to the client's needs and act accordingly.
*Two‐week interval with practical assignment*		
2	Collaborating and shared decision making	The participant: can engage in dialogue with the client and significant others to determine where the client needs support in making health‐related choices and where they can manage independently. is aware of whom to collaborate with to promote the client's health. knows who can be involved in carrying out actions outlined in the client's care plan.
*Two‐week interval with practical assignment*		
3	Promoting healthy living	The participant: is aware of how to promote a healthy lifestyle to prevent health problems. knows what a healthy lifestyle entails.

### 2.3 Participants and Recruitment

Participants were adults with at least one year's experience working professionally as a support worker for people with intellectual disabilities in residential or day‐activity settings. They were recruited through (1) the network of the academic collaborative ‘Stronger on your own feet’, a collaboration between six Dutch care‐provider organisations for people with intellectual disabilities and an academic institute and (2) the national professional association in the Netherlands (BPSW), which assists support workers in advancing their careers through advocacy for the profession and knowledge exchange. Information about the training and the research was shared via social media and the newsletters of the academic collaborative and the professional association. Participants in the two pilot groups were offered the training free of charge. An online registration process directed potential participants to a registration form. Participants could enrol in one of the two available training groups, which were scheduled on different days. Upon registration, they were emailed a consent form and an information leaflet about participating in the training and the research project, with the option to ask further questions. Commitment to attending all training sessions and data collection occasions was required for inclusion. Participants received email confirmation of their participation. The researcher (KN) collected signed consent forms either via email or on the first day of the training.

In total, 30 persons enrolled in the two pilots. However, two participants were absent at the start of the first group's training and another two failed to attend all data collection occasions because of illness and for unspecified reasons. Two groups of 12 and 14 participants (*N* = 26) completed the three‐day training programme and participated in associated data collection, as described in Table 2.

**TABLE 2:** Descriptives of participants in two training pilots for the TRAIN‐ID programme about health support for people with intellectual disabilities.

Focus group number	Participant number	Age	Female/male	Experience working with people with intellectual disabilities in years	Work setting: residential (R), day‐activity centre (D) or ambulatory (A)	Educational background: social care (SC), nursing (N) or other (O)	Intellectual disability level of client with whom they work
*Training Group 1*							
1	1	29	F	6	R	SC	Mild–moderate
	2	58	F	16	R	SC	Mild–profound
	3	51	M	5	R and D and A	SC, O	Mild–profound
	4	45	F	22	R	SC, N	Mild
	5	52	F	33	R	SC	Mild–profound
	6	50	F	30	R	SC	Mild
Mean *(sd)*		47.5 *(9.9)*		18.7 *(11.8)*			
2	7	41	F	16	R	SC	Mild–moderate
	8	36	M	5	R and D	SC	Mild
	9	38	M	12	D	SC	Mild–profound
	10	44	F	20	R	SC	Mild
	11	36	F	17	D	Unknown	Severe
	12	49	F	29	R	N	Moderate
Mean *(sd)*		40.7 *(5.1)*		16.5 *(8.0)*			
3	*Training Group 2*						
	13	57	F	3	R	SC, O	Profound
	14	49	F	27	R	SC	Moderate
	15	28	F	8	R and A	N	Mild–moderate
	16	55	F	20	R and A	SC	Mild
	17	27	F	8	R	SC	Mild–moderate
	18	57	F	2	R	SC, O	Mild–profound
	19	48	F	7	R	SC, O	Mild
Mean *(sd)*		44 *(13.2)*		12 *(9.4)*			
4	20	57	F	20	R	SC	Mild
	21	35	F	4	R and A	SC, O	Mild
	22	27	F	5	R	SC	Moderate–severe
	23	29	F	8	R	SC	Mild–profound
	24	53	F	26	R	SC	Mild–profound
	25	24	F	3	R	SC	Mild–profound
	26	59	F	1	R	SC, O	Severe
Mean *(sd)*		37.8 *(14.6)*		7.8 *(9.2)*			

### 2.4 Data Collection

Data were collected in the Netherlands during May and June 2024. Data collection guides (provided in the Supporting Information as Appendix A) were developed by the research team (K.N., K.B., F.B., J.N., G.L.) in collaboration with a social work lecturer (E.vO.) and an education specialist with expertise in the Kirkpatrick model. The guides were informed by the Kirkpatrick framework to structure the questions and ensure that the questions addressed both participants' satisfaction with the training and the perceived impact on the delivery of health support in everyday practice. Questions were organised around the Kirkpatrick levels, covering participants' evaluation of the training (reaction), perceived learning outcomes such as new awareness, knowledge or skills (learning), changes in professional behaviour in daily work (behaviour) and perceived impact on the health support provided to clients (results). The semi‐structured questions encouraged participants to describe concrete experiences and provide examples from practice. To ensure that the FGD moderators were familiar with the procedure and to promote consistency across sessions, the FGD guide was pilot‐tested at a procedural level with colleagues of the moderators (K.N., M.T., A.K., S.Z.). No modifications were made following the pilot‐testing.

#### 2.4.1 During the Training

At the end of each training day, participants were asked to reflect on their experiences with the training (Appendix A1). Participants answered individually on sticky notes how they experienced this training day and what they would share with their professional team about this day. These written reflections were briefly discussed in a plenary session, after which researcher K.N. collected the written reflections to include in data analysis.

#### 2.4.2 Immediately Post‐Training

At the end of the third training day, participants joined a one‐hour FGD with two moderators per FGD (K.N. and research assistants) to reflect on and discuss their experiences of the training (Appendix A2). Within each training group, participants were randomly divided into two subgroups of six to seven persons, resulting in four FGDs in total. Before the discussion, each participant rated the training on a scale from 1 to 10 and wrote the score on a sticky note. These ratings were collected to calculate a descriptive overview of participants' overall evaluation. Participants were then instructed to provide a written explanation for their rating. The moderators collected these notes as participants were writing, grouped them by identifying recurring words or themes and used these groups to guide a semi‐structured group discussion. The discussion was guided by the content of the notes and questions addressing the perceived usefulness of different training components, what participants had learned, how they expected to apply this in daily work and what impact they anticipated on the health support provided to clients. All FGDs were audio‐recorded, transcribed verbatim and pseudonymised before analysis.

#### 2.4.3 Three Months Post‐Training

Approximately three months post‐training, telephone interviews were conducted to explore participants' experiences with the training programme and its perceived impact on health support in daily care for people with intellectual disabilities. Interviews followed a semi‐structured guide (Appendix A3), starting with a rating of the training programme on a 1–10 scale and an explanation of this rating and then asking follow‐up questions. Interview questions explored participants' evaluation of the training, perceived learning outcomes, changes in daily work practices and perceived impact of these changes on clients or organisational care processes. All interviews were conducted by the main researcher (KN), who took detailed manual notes during each interview to compile an interview summary, an approach that previous studies suggest can provide sufficiently rich data for applied qualitative research (Hill et al. 2022; Rutakumwa et al. 2020).

### 2.5 Data Analysis

The aim of this study was to gain insight into support workers' experiences with the training programme and its perceived impact on health support. The focus was not on comparing participants or data collection occasions. Therefore, all data (consisting of the written reflections, FGD transcripts and interview summaries) were analysed as a unified set. Using the Kirkpatrick framework enabled a systematic examination of training outcomes across different levels of impact, from participants' experiences and learning to perceived changes in behaviour and results (Kirkpatrick and Kirpatrick 2011). The data were analysed according to the steps of template analysis (King et al. 2017), offering a structured yet flexible approach to data interpretation (King and Brooks 2016). The Kirkpatrick framework informed the development of the initial coding template, enabling a systematic examination of training outcomes across different levels of perceived impact. This approach supported consistent coding across data sources while allowing refinement of themes during the analytic process.

The numerical ratings (on a 1–10 scale) provided immediately after the training were used to capture participants' reaction and as a starting point for the discussion. Mean scores and standard deviations were calculated to provide an overall indication of participants' evaluation of the training (Kirkpatrick Level 1: Reaction). The written reflections, FGD transcripts and interview summaries were analysed supported by Atlas.ti 24.0.0 software (Friese et al. 2018).

First, the research team (K.N., K.B., J.N., G.L.) developed an initial template that included the Kirkpatrick levels and clear criteria for the type of statements to be coded within each level (see Table 3). Second, one FGD transcript was used as a pilot dataset for the initial coding process. This transcript was independently coded by two researchers (K.N. and A.K.), who focused on identifying relevant text fragments and associating them with the corresponding Kirkpatrick levels. Descriptive notes were added to the dataset to capture initial interpretations, researchers' doubts about the codenames and connections between codes. This was followed by discussion and consensus‐building among the researchers (K.N., A.K., K.B., J.N.) to refine the definitions within each level. Third, the refined template was tested on three interview summaries coded independently by K.N. and A.K. During this preliminary coding phase, new codes were introduced to capture topics under each assigned Kirkpatrick level. Fourth, codes were clustered into groups within the Kirkpatrick levels, resulting in an expanded template with subthemes. The definition of behaviour was broadened to include both enacted and intended behaviour. This decision was informed by the finding that the three‐month follow‐up captured early stages of learning being applied in practice. The fifth and final step consisted of applying the revised template to the remaining dataset, including the other FGD transcripts, interview summaries and written reflections. K.N. coded the remaining data and connected them to the (sub)themes in the template. The template was iteratively refined throughout this step, with the subthemes being revised based on ongoing discussions and reflections between K.N., K.B., J.N., G.L. The initial and the final template are presented in Table 3. Alongside the template analysis, K.N. examined the data for text fragments that demonstrated relationships between Kirkpatrick levels, enabling the identification of pathways linking reaction, learning, behaviour and perceived results.

**TABLE 3:** Initial and final templates, based on Kirkpatrick levels, for data analysis of the TRAIN‐ID training programme about health support for people with intellectual disabilities.

Initial template		Final template
Main theme	Criteria	Theme, subtheme and description
K1_Reaction	Satisfaction Engagement Relevance Format	*Reaction* K1_Overall experience Overall impressions and general experiences of the training programme K1_Training content Perceptions of the relevance, clarity and usefulness of the training content K1_Training format Experiences with the structure, methods and delivery format of the training K1_Training communication Experiences with communication before and during the training programme
K2_Learning	Knowledge Insights Awareness Skills Confidence	*Learning* K2_Health problems of people with intellectual disabilities Learning common health conditions in people with intellectual disabilities and differences K2_Health signals Learning underlying signs that may indicate physical or mental discomfort or health issues K2_Health assessment Learning to observe, interpret and assess health‐related signals, including atypical presentations K2_Lifestyle Learning about lifestyle factors related to the health of people with intellectual disabilities K2_Communication Learning communication approaches for discussing health with clients and other stakeholders K2_Support workers' professional role Learning about the role that support workers can play in support clients with their health
K3_Behaviour	Application of learning Support workers' behavioural change	*Behaviour* K3_Discussing health with clients Perceived/intended changes in how health‐related topics are discussed between support workers and clients K3_Following up Perceived/intended changes in how support workers follow up on health concerns in daily practice K3_Interacting with healthcare professionals Perceived/intended changes in interaction with healthcare professionals K3_Using training materials Application and sharing of training materials and tools within the work setting
K4_Results	Change in: Care quality Clients' health Clients' or colleagues' behaviour	*Results* K4_Care situation of client Perceived impact on the organisation and delivery of health support in daily care K4_Behaviour client Perceived impact on clients' health‐related behaviour K4_Interdisciplinary health support Perceived impact on teamwork and communication among colleagues

## 3 Results

We present the results per template level. Additionally, we describe pathways whereby the levels interconnect, illustrating how insights gained from the training (Level 2) were applied in daily practice (Level 3) and how these behaviours were perceived to impact health support (Level 4).

### 3.1 Kirkpatrick Level 1: Reaction

Participants evaluated the training positively, with a mean score of 6.9 (SD 2.1) on a scale of 1–10. Their appreciation concerned the variety in learning activities and how the training closely aligned with the participants' daily practice. The trainer's expertise and experience as an intellectual disability physician and the involvement of experts‐by‐experience were regarded as meaningful and informative. Participants also valued the interactive training format, with well‐organised breaks and catering and sufficient time for dialogue and exchanging experiences and dilemmas. Suggestions for improvement focused on the organisation of the training. Given the travel time involved, participants preferred full‐day sessions over half‐day sessions as this would better justify their time investment and allow for more in‐depth engagement. Additionally, participants stated that clearer communication about content was needed prior to the training, as many initially expected lifestyle‐focused training, with mismatches between expectations and content. Despite this, the focus on health characteristics of people with intellectual disabilities was experienced as unexpectedly valuable.

### 3.2 Kirkpatrick Level 2: Learning

Learning concentrated on the topics: Health problems of people with intellectual disabilities, Health signals, Health assessment, Lifestyle, Communication and Support workers' professional role. Participants differed in what they reported learning from the training, ranging from knowledge to the acquisition of awareness or skills.

Understanding common health problems in people with intellectual disabilities, their causes and how these problems may present was reported as an important learning outcome, contributing to awareness of health differences between people with intellectual disabilities and the general population. Learning about recognising health signals was identified as contributing to understanding how physical health problems may become visible through behavioural changes. This was expressed as being able to identify subtle signs, such as changes in movement, appetite or sleep, that might indicate underlying health concerns. Practicing purposeful observation and discussing observations were experienced as helpful exercises for assessing health concerns. This enabled participants to look beyond first impressions, to consider alternative explanations for what they observed and to think through appropriate next steps. For example, participants described having learned to reinterpret situations that were previously seen as routine by considering whether behavioural or physical changes might reflect underlying health concerns requiring further attention, rather than assuming that routine responses (e.g., giving a painkiller) would be sufficient.

For the lifestyle topic, identified learning outcomes included understanding the impact of lifestyle on health and confidence in addressing clients' (un)healthy lifestyle. In addition, both participants and an expert‐by‐experience presented successful cases of lifestyle change among people with intellectual disabilities that motivated engagement in lifestyle support. Participants valued learning communication techniques for explaining health information in accessible ways, for checking comprehension among clients and for motivating their clients to make healthier choices. The relevance of involving experts‐by‐experience in the training programme became evident, as participants described how these contributions increased their awareness of and skills in, involving clients in discussions about their health. Learning about theory and hands‐on practice relating to motivational interviewing was identified as insufficient in the current version of the training programme.

Learning about the care network and awareness of support workers' important role in managing their clients' health in everyday contexts was viewed as boosting confidence and providing valuable insights into how to navigate the complexity of the broader care network. For example, a deeper understanding of healthcare professionals' concerns helped participants learn how to collaborate more effectively. Participants reported learning that their unique insight into the client's daily life, combined with the medical expertise of healthcare professionals, created a more effective approach to care.

### 3.3 Kirkpatrick Level 3: Behaviour

Behavioural change was described across four topics: Discussing health with people with intellectual disabilities, Following up on health concerns, Interacting with healthcare professionals and Applying training information and tools. Within each topic, the following sections describe both intentions to change behaviour and examples of behaviour that had already been applied in practice.

Participants intended to use communication techniques from the training to better acknowledge clients' experiences and to create a sense of their being heard during conversations about health. Participants also described actual behavioural changes, such as applying these techniques in practice when discussing health‐related issues with clients. For example, at moments of disagreement or tension, strategies introduced by the expert‐by‐experience were used to reflect on the client's perspective and support more constructive interactions.

Intentions about following up health concerns included being more alert to changes in clients' well‐being and first exploring alternative explanations or follow‐up steps. Practical tools introduced during the training, such as checklists for changes in behaviour related to physical health problems, were mentioned as helpful for explaining unusual behaviour by clients. Behavioural changes already applied in practice included more frequent exchange of observations with colleagues and more discussion of complex cases, reflecting a more proactive approach to follow‐up.

The trainer's emphasis on the importance of support workers' role in health support was experienced as strengthening participants' confidence to act on this responsibility, motivating them to raise concerns earlier and more assertively. Participants described intentions to communicate more proactively in their interactions with healthcare professionals, particularly when sharing observations and perspectives on a client's health status. This confidence also led to concrete behavioural changes, such as asking follow‐up questions and not immediately accepting reassurances when concerns were initially not taken seriously. Participants indicated that, prior to the training, doubts about healthcare professionals taking them seriously had hindered such communication.

Practical materials from the training, such as visual aids demonstrating nutritional information, were used to support accessible conversations about health. Participants also intended to share tools and information with colleagues, including conducting experiential exercises together to simulate, for example, how it feels to have one's teeth brushed by someone else. Behavioural changes already implemented included actively sharing training materials within teams, for instance by uploading materials to online platforms, thereby extending individual learning to team‐level practice.

Despite these intended and actual behaviour changes, participants described situations in which applying training information and tools proved difficult. Contextual factors such as high workload, limited time and staffing constraints influenced behaviour change in health support. These factors shaped whether intended behaviour could be translated into changes in daily practice.

### 3.4 Kirkpatrick Level 4: Results

Changes in participants' actions following the training were perceived to result in varying client or organisational outcomes. The results included Care situation of client, Clients' behaviour and Interdisciplinary health support. The following examples illustrate pathways through which insights gained from the training (Level 2) were applied to different extents in daily practice (Level 3), resulting in either perceived changes or no noticeable changes in health support (Level 4). The pathway examples in Figures 1, 2, 3, 4 were based on individual interview summaries and paraphrased. They reflect participants' reported experiences and are not direct quotations.

**FIGURE 1:**
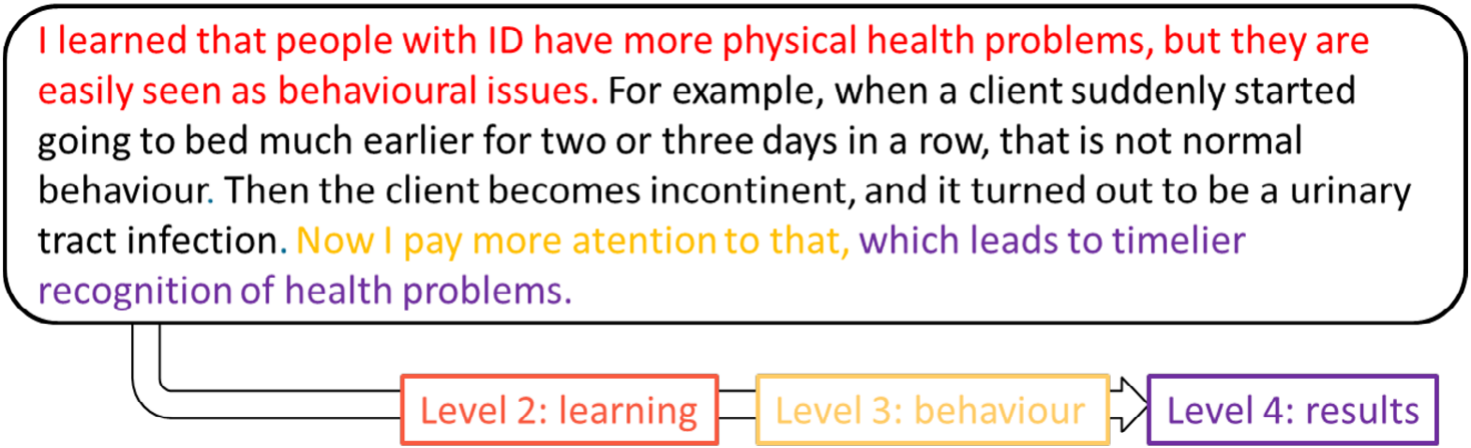
Example of a pathway illustrating result in Clients' care situation (Interview participant 7).

**FIGURE 2:**
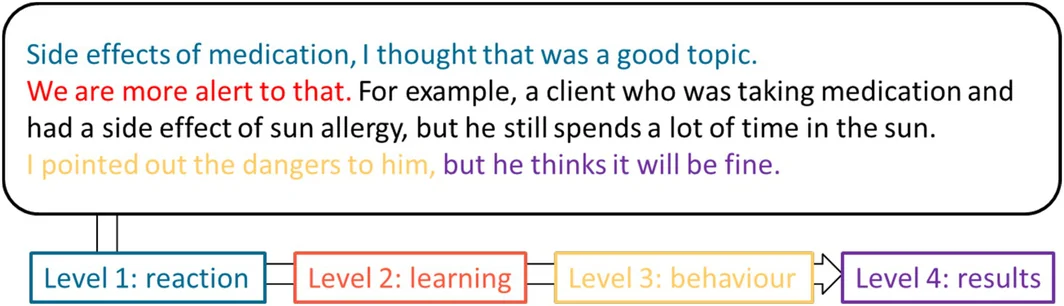
Example of a pathway illustrating result in Client behaviour (Interview participant 8).

**FIGURE 3:**
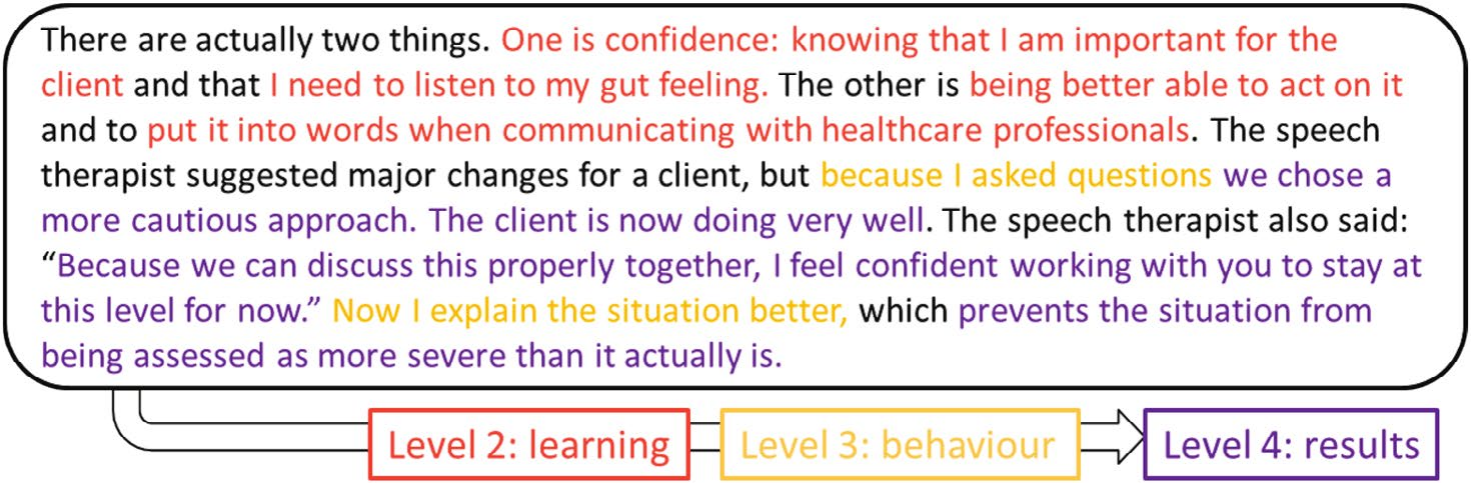
Example of a pathway illustrating result in Interdisciplinary health support (Interview participant 14).

**FIGURE 4:**
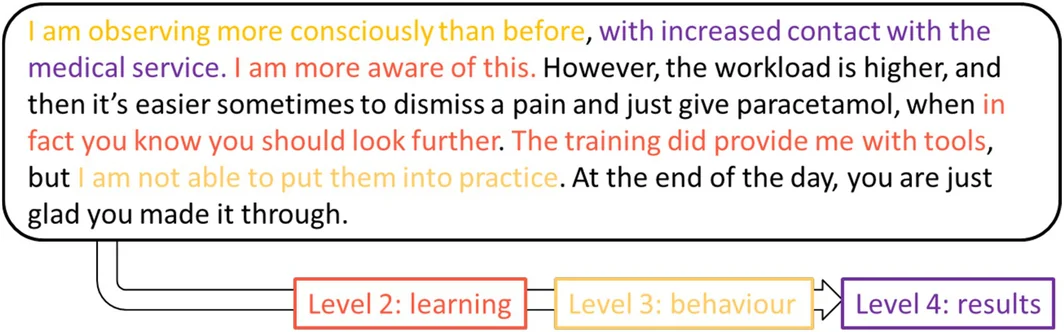
Example of a pathway illustrating the work context in relation to the training (Interview participant 16).

Changes in clients' care situation were explained through improved day‐activity programmes, re‐evaluation of medication, healthier reward practices and more effective use of reporting systems. Participants also described earlier identification of health problems, such as swallowing difficulties or urinary tract infections. Figure 1 presents an example illustrating how such changes were perceived in daily care.

Knowledge and awareness about the health of people with intellectual disabilities (Level 2) led to changes in observation and interpretation (Level 3), which were perceived to contribute to earlier identification of health problems and improvements in the client's care situation (Level 4).

Participants also reported more actively supporting people with intellectual disabilities in making healthier choices, for example when grocery shopping or snacking. In some cases, this resulted in healthier client behaviour, whereas in others no behavioural change was perceived, despite participants' efforts. These situations were often attributed to clients' limited capacity to anticipate consequences or to difficulties in changing established routines. Figure 2 demonstrates a pathway to a perceived result in a client's behaviour.

This example demonstrates a pathway whereby knowledge and alertness regarding health risks (Level 2) prompted active communication about health risks (Level 3), without this translating into perceived behavioural change by the client (Level 4).

According to participants, confidence in their important role and in their ability to communicate health signals helped them seek timely support in challenging situations. This often led to perceived improvements in communication and collaboration between participants and healthcare professionals. Participants described being more actively involved in discussions about clients' health and care decisions. Figure 3 presents an example of how interdisciplinary collaboration was experienced in practice.

Support workers' confidence in their professional role and communication skills (Level 2) facilitated more active and critical engagement with healthcare professionals (Level 3), contributing to informed decision making and perceived positive outcomes for the client (Level 4).

Additionally, participants reported contextual factors that limited perceived outcomes, including clients' cognitive limitations and reliance on routines, high workload, staffing shortages, time pressure and unclear responsibilities within care teams. These factors were described as constraining the application of new insights and practices in daily care. Figure 4 illustrates how such contextual conditions shaped the translation of learning into practice.

This example illustrates how increased awareness of health‐related signals (Level 2) was associated with changes in a participant's observing behaviour (Level 3), but contextual constraints hindered further behaviour change (Level 3) or perceived outcomes (Level 4).

Together, these pathways show participants' examples of the relationship between learning, behaviour and perceived results, which is not straightforward in everyday care practice. In some situations, insights gained during the training were perceived to translate into changes in participants' actions that were associated with better decision making and client outcomes, particularly when there was room for dialogue and collaboration with healthcare professionals. In other situations, participants reported that learning resulted mainly in increased awareness or alertness, without leading to behavioural change or perceived outcomes. Such situations often arose from contextual factors such as high workload, staff shortages or the complexity of clients' characteristics.

## 4 Discussion

This study provides insights into support workers' experiences with the TRAIN‐ID training programme and their perceived impact on health support for people with intellectual disabilities. The TRAIN‐ID training programme was designed to broaden support workers' understanding of health needs and health inequities related to intellectual disability and it positions support workers as key actors in recognising and following up health concerns in daily care. This study shows how training outcomes depend not only on what support workers learn, but also on how support workers can implement what they have learnt, considering their role within care systems and the opportunities available within care organisations.

The training was experienced as relevant, as it aligned closely with support workers' everyday practice. Participants particularly valued the practical orientation of the training and the opportunity to reflect on real cases and dilemmas from daily care. Rather than perceiving the training as passive knowledge transmission, support workers described it as an active learning process that combined reflection, discussion and practical exercises. Active learning approaches, such as case‐based learning, reflective discussion and peer exchange, are widely recognised as effective in professional education because they support the integration of theoretical knowledge with daily practice (Heck et al. 2023; Kolb 2013; Eraut 2013). These findings highlight the importance of training in supporting the integration of learning into everyday practice. By repeatedly connecting theoretical insights to support workers' own cases and encouraging reflection on daily dilemmas, the training supported the integration of new perspectives on health support rather than merely adding isolated knowledge or skills. Similar findings have been reported in social work education, where reflective learning approaches contribute to professional identity formation and sustained integration of learning into practice (Beddoe 2013).

Within this active learning approach, the involvement of experts‐by‐experience added an extra dimension by bringing lived experience of intellectual disability into the training environment, enabling support workers to reflect directly on their communication skills. Previous research has shown that including lived experience in professional education can enhance empathy, improve communication skills and deepen understanding of the everyday realities of people with disabilities (Happell et al. 2022; Kerry et al. 2023). In addition to learning from lived experience, support workers valued the opportunity to exchange experiences with colleagues from different care settings. Such peer learning has been identified as an important mechanism through which professionals interpret new knowledge, compare practices and collectively reflect on challenges in their work (Smith et al. 2025; Bingen et al. 2024). Beyond these learning processes, support workers described changes in how they interpreted health‐related situations in daily care. Experiential learning and involvement of experts‐by‐experience support the transfer of learning into practice by linking new knowledge to concrete situations and encouraging reflection from the client's perspective (Happell et al. 2020). These approaches can help bridge learning and application, although their specific contribution to behavioural change was not examined in this study.

Learning involved awareness of health risks among people with intellectual disabilities and a clearer understanding of support workers' contribution to health support within interdisciplinary care. The training programme encouraged support workers to have a more attentive and objective approach to subtle changes in their clients' behaviour and daily functioning that may signal underlying health problems. This shift in interpretation is particularly relevant given the well‐documented phenomenon of diagnostic overshadowing, in which health problems are misattributed to behavioural or disability‐related causes (Ervin et al. 2014; Salvador‐Carulla et al. 2015). When support workers adopt a more reflective and health‐oriented lens in their daily observations, the TRAIN‐ID training programme may support earlier recognition of health concerns and more appropriate follow‐up.

Although support workers described increased awareness of health signals, this learning did not automatically translate into action. Support workers often linked acting upon their observations to their perceived confidence in communicating health concerns to healthcare professionals. This finding aligns with previous studies showing that professionals are more likely to apply newly acquired skills when training strengthens their sense of professional authority (Maine et al. 2017; Nota et al. 2007). Role strengthening may therefore reduce support workers' previously reported hesitation to speak up, which has been linked to perceived power imbalances or doubts about professional expertise (Ambrose‐Miller and Ashcroft 2016).

A key insight from this study is that training impact unfolded through different pathways for each support worker in relation to learning, behavioural change and perceived impact. The outcomes showed variety in how support workers translated learning into behaviour change. Factors such as workload, staffing levels, time pressure and the complexity of clients' support needs influenced whether support workers were able to act upon their learning. Similar context‐dependent training effects have been reported in intellectual disability care and broader healthcare settings, where organisational conditions strongly influence the translation of training into practice (Rose and Gallivan 2019; Kersten et al. 2025, 2022; Bartlett 2001; Lyng et al. 2022). Importantly, the absence of observable behavioural change should not necessarily be interpreted as ineffective learning. In complex care environments, outcomes such as increased awareness, confidence and professional judgement may represent meaningful intermediate steps towards improved care.

Training effects are stronger when supported by organisational structures that enable professionals to apply new knowledge and skills (Eccles et al. 2009; Patey et al. 2018). Embedding training within team routines, involving supervisors and aligning training content with organisational practices are therefore necessary to ensure sustained improvements in health support across care settings (Aruldoss et al. 2022). Future research could examine how such implementation strategies influence the uptake and impact of training programmes, such as TRAIN‐ID, across different organisational contexts.

Taken together, the findings have implications for both practice and research. For practice, training programmes are advised to combine practice‐oriented learning with opportunities for reflection, peer exchange and involvement of experts‐by‐experience. Organisations should create conditions that support the application of learning in daily care. Findings from the pilot also provide directions for further refinement of TRAIN‐ID, including balancing time investment and training content, clear communication and sufficient skills practice, while retaining its practical relevance, health‐related focus and attention to support workers' professional role. For research, longer‐term evaluations are needed to examine whether learning and early behavioural changes are sustained and translate into client outcomes. We did not investigate how organisational conditions or training format relate to training impact, which can be topics for future research.

## 5 Strengths and Limitations

A key strength of this study is the diversity of the participant group, including support workers with varying educational backgrounds, levels of experience, age and work settings. This heterogeneity enriched the data and allowed for a broad range of perspectives on training experiences and perceived impact across different intellectual disability care contexts in the Netherlands. In addition, the qualitative design enabled an in‐depth exploration of support workers' experiences, learning processes and perceived impact of the training programme in daily practice. The use of multiple data sources collected at different time points further strengthened the study. Written reflections captured participants' immediate responses to the training, while focus groups and follow‐up interviews provided broader reflections on learning and its application in practice. This approach helped identify contextual influences on learning and behavioural change, as well as outcomes such as increased understanding of support workers' role in health support that might have been overlooked in evaluations relying solely on predefined quantitative measures. The Kirkpatrick framework provided a useful structure for examining participants' reactions, learning, behaviour and perceived results, as well as how learning was perceived to translate into professional behaviour and health support in daily practice. This study thereby contributes to the limited scientific evidence base on training effectiveness in intellectual disability care.

This study also has limitations. The predefined levels of the Kirkpatrick framework may have focused attention on findings that fit these categories, while other processes outside the framework may have received less attention. This risk was partly addressed by using template analysis flexibly, allowing new codes and themes to emerge from the data, including contextual factors influencing the transfer of learning into practice. Also, the follow‐up period of three months may have captured intentions and early changes rather than sustained behavioural change or longer‐term outcomes. A longer follow‐up period would provide greater insight into whether learning is translated into enduring practice. Furthermore, the fact that the study was conducted within the Dutch high‐resource care context may limit its generalisability to other countries with different healthcare systems, supporting roles and organisational structures.

## 6 Conclusion

This study suggests that the TRAIN‐ID training programme can play a role in strengthening support workers' contribution to everyday health support for people with intellectual disabilities. Using the outcomes of this study to adapt and enhance the TRAIN‐ID training programme, an evidence‐informed training programme can be developed that equips support workers to prevent, identify and follow up on health concerns relating to the clients that they support. Training programmes that recognise and sustain support workers' vital role in reducing health inequities can help strengthen this role, reduce educational gaps and improve support workers' ability to meet the complex health needs of people with intellectual disabilities.

## Author Contributions

**Kim Nijhof:** conceptualisation, methodology, validation, formal analysis, investigation, data curation, writing – original draft (lead), visualisation, project administration. **Fleur H. Boot:** conceptualisation, methodology, validation, formal analysis, data curation, writing – review and editing. **Kirsten E. Bevelander:** conceptualisation, methodology, validation, formal analysis, data curation, writing – review and editing. **Elske van Oostaijen:** conceptualisation, methodology, formal analysis, writing – review and editing. **Jenneken Naaldenberg:** conceptualisation, methodology, validation, formal analysis, data curation, writing – review and editing, supervision. **Geraline L. Leusink:** conceptualisation, methodology, validation, formal analysis, writing – review and editing, supervision.

## Funding

This work was supported by ZonMw, the Netherlands Organization for Health Research and Development (grant number 80‐85300‐98‐19110).

## Ethics Statement

The Ethical Review Board of Radboud University Medical Center assessed this study (Registration number 2024‐17091) and waived the requirement for a full review according to the Dutch Medical Research with Human Subjects Law (Wet Medisch‐wetenschappelijk Onderzoek met mensen (WMO)). We followed the ethical principles of the Declaration of Helsinki and General Data Protection Regulations.

## Consent

All participants provided written informed consent prior to participation in the study.

## Conflicts of Interest

The authors declare no conflicts of interest.

## Supporting information


**Appendix A.** Data collection guide.

## Data Availability

The dataset contains identifiable information and was not collected for public sharing. No consent for open access was obtained. Therefore, data are not publicly available. Supporting materials (e.g., interview guide, coding scheme) are provided in the Radboud Data Repository (https://doi.org/10.34973/g9hv‐zv18).
