# Supplementary material for: From Learning to Practice: Support Workers' Experiences With the TRAIN‐ID Training Programme to Strengthen Health Support for People With Intellectual Disabilities
Source: J Appl Res Intellect Disabil. 2026 Oct 20;39(5):e70326. doi: 10.1111/jar.70326 (PMC13682912; doi:10.1111/jar.70326)
Supplement: Supplementary file 1 [file JAR-39-e70326-s001.docx]

**Appendix A: Data collection guide**

**A1**

| **Data collection occasion** | **Method** | **Procedures** |
| --- | --- | --- |
| **During the training** | Feedback notes | Before leaving the training room at the end of each training day, participants responded to two questions:   - How did you experience today? - What will you share with your team about today?   Responses were written on sticky notes and placed on two central boards in the training room. Responses were collected anonymously by the researcher. |
| **A2** | | |
| **Directly after completion of the training** | Focus group discussions | First, the moderators (KN or MT) plenarily explained the purpose and instructions for the focus group discussion. Then, the participants were asked to rate how they experienced the training programme on a scale from 1 to 10, and they wrote this on sticky notes. Participants were then invited to explain their answers and instructed to take ten minutes to write short answers, each on a separate sticky note. While the participants were writing, the first (KN or MT) and second moderators (AK or SZ) quietly collected the sticky notes, softly discussed their categorisation, grouped them based on recurring words or themes, and placed them on a wall in the same room. After ten minutes, the participants had to stop writing and had five minutes to walk alongside the wall and read all sticky notes. The moderator started a group discussion, guided by the content of the sticky notes and questions guided by the Kirkpatrick model:   - Which section or element of the training programme appealed to you most and why? - Which section of the training programme was the most or the least informative? What could make the training more informative? - To what extent do you think you can apply the knowledge and skills you learnt during this training in your daily job? - How will you implement these new skills and knowledge into your daily job? - What do you think the impact of the training programme is or will be on the health support of the clients whom you support? - Open question: general comments on the training programme. |
| **A3** | | |
| **Three months after the training** | Telephone interviews | The researcher (KN) conducted all interviews and took notes of participants’ responses to her questions. The researcher asked the following questions and asked follow-up questions to gain additional insights or explanations.   - How relevant was the training programme if you look back? Please explain your answer. - What new awareness, knowledge, or skills have you acquired through the training programme? - What do you do differently in your daily job since you followed the training programme? - What does your client or the organisation for which you work notice as a result of you having followed the training programme? - Open question: General comments on the training. |
